# Effect of Nigella sativa Versus Wheat Germ Oil on the Healing of Traumatic Ulcers in Albino Rats

**DOI:** 10.7759/cureus.52432

**Published:** 2024-01-17

**Authors:** Omar M Mohamed, Ghada A ElBaz, Enas M Hegazy, Yousra S Helmy

**Affiliations:** 1 Pediatric and Preventive Dentistry and Dental Public Health Department, Faculty of Dentistry, Suez Canal University, Ismailia, EGY; 2 Oral Biology Department, Faculty of Dentistry, Suez Canal University, Ismailia, EGY

**Keywords:** nigella sativa oil, wheat germ, nigella sativa, pediatric dental trauma, traumatic oral ulcers

## Abstract

Background and objective: *Nigella sativa* (NS) oil has been used as an ointment for relief from abscesses, nasal ulcers, orchitis, eczema, and swollen joints. The nutritional and biological values of wheat germ oil (WGO) are imperative points for testing its wound healing properties in traumatic ulcer. The aim of the study was to evaluate and compare the ability of NS versus WGO in promoting the healing of induced traumatic ulcer in albino rats clinically and histologically.

Materials and methods: This study was carried out after the approval of the Research Ethics Committee (REC) of the Faculty of Dentistry, Suez Canal University, in Ismailia, Egypt, on 60 albino rats with induced labial ulcer according to calculated sample size. All animals were anaesthetized with an intraperitoneal injection of 10% ketamine. The ulcer was produced on the labial mucosa corresponding to the midline between the lower two incisors of each rat. After induction of the ulcer, rats were randomly divided into four groups according to the treatment medicament: Group A (negative control group): 15 rats which remained without treatment; Group B (positive control): 15 rats which received daily a topical application of 1 ml of cetylpyridinium chloride (CPC) and lidocaine gel; Group C (NS group): 15 rats which received a daily topical application of 1 mm of NS oil painted by a brush covering the whole area of the ulcer; and Group D (WGO group): 15 rats which received 1 mm of WGO. The ulcers were measured using a digital caliper and were recorded using a digital camera at days 0, 3, 7, and 9, the largest (D) and smallest (d) diameters of the lesion were recorded, and the ulcer area was calculated using the following formula: A=π×D/2×d/2. Tissue samples were taken for histological examination, and the labial mucosa was dissected out and embedded in paraffin wax blocks. The blocks were cut with microtome to obtain sections of 4-5 μm thickness to be stained with hematoxylin and eosin stain and Masson's trichrome stain. All sections were examined under a light microscope, and the presence of inflammatory cells and collagen tissue remodeling were evaluated.

Results: Within the control group, there are statistically non-significant changes in the mean of the surface area of ulcer when comparing changes in 10 rats who survived till the seventh day and inflammatory cell count when comparing changes in five rats who were sacrificed at the seventhday. There was a significant decrease in surface area and inflammatory cell count in five rats who survived till the ninth day. Within the WGO group only, all survived rats had healed ulcer at the ninth day. There is a significant decrease in inflammatory cell count in five rats who survived till the ninth day.

Conclusion: WGO was significantly more effective in the treatment of animal-induced ulcer compared to NS oil or CPC and lidocaine oral gel.

## Introduction

Ulcer is a rupture or discontinuity in a body membrane that prevents the organ in question from functioning normally. One of the most frequent causes of consultations for oral medicine is oral ulcers. It is commonly described as damage to the oral mucosa's lamina propria and epithelium that results in the oral mucosa's discontinuity [1].

In general, ulcers can be categorized as either acute (short-term) or chronic (long-term). It can be difficult to diagnose mouth ulcers; therefore, it's crucial to think about other possible diagnoses. The length, related symptoms, pattern of incidence or recurrence, and related systemic illnesses should all be considered while obtaining a history [2].

Typically, traumatic injuries involving the oral cavity may lead to the formation of surface ulcerations. These wounds might be the consequence of subsequent bite or mechanical trauma, such biting oneself while talking or sleeping. There may also be thermal, electrical, or chemical assaults [3].

With a long history, *Nigella sativa* (NS) has been used as a herbal medicine to treat a wide range of conditions, such as infertility, fever, rheumatism, bronchitis, asthma, chronic headaches, migraines, dizziness, chest congestion, paralysis, hemiplegia, back pain, dysmenorrhea, obesity, diabetes, and infections and inflammation. Furthermore, NS oil has been used topically to treat dermatitis, nasal ulcers, abscesses, and swollen joints [4].

The enhanced fibroblast proliferation, subsequent collagen production, and stimulation of angiogenesis are the primary mechanisms via which NS promotes skin wound healing. It has also been shown that NS lowers the amount of bacterial infection, tissue damage, and white blood cell presence [5].

It is well known that wheat germ (WG) is a nutrient-dense raw material that may be used to make food products or eaten on its own. The benefits of wheat germ oil (WGO) are many. It is used in the culinary, cosmetic, farming, and medical industries. Its uses include the manufacturing of dietary supplements and vitamins, the creation of animal feed, the biological control of insects, and the treatment of cardiac and circulatory diseases [6,7].

Many components are included in WGO, including flavonoids, glutathione, and oleic, palmitic, stearic, arachidonic, and linoleic fatty acids. This is why reports of its antioxidant potential have surfaced. Additionally, WGO's unsaturated fatty acids function as precursors to prostaglandin hormones, which lessen inflammation and aid in healing. *Streptococcus epidermidis* and *Pseudomonas aeruginosa* were the microorganisms against which WGO showed antibacterial action. Several skin ulcers have been linked to these organisms, which have also been identified as a frequent source of postoperative infection. In order to evaluate WGO's ability to cure wounds in traumatic ulcer patients, it is essential to consider both its nutritional and biological qualities and its frequent use in skin care products [8].

The current study was designated to evaluate and compare the ability of herbal medicine elements such as NS and WGO versus conventional synthetic treatment (cetylpyridinium chloride (CPC) and lidocaine gel) in promoting the healing of induced traumatic ulcer in albino rats clinically and histologically.

## Materials and methods

The current research experiment was performed in the animal house of the Faculty of Dentistry, Suez Canal University, in Ismailia, Egypt, after the approval of the Research Ethics Committee (REC) of the same institution (approval number: 151/2018).

Materials

Table 1 presents the materials that were used in the study including NS oil, WGO, and CPC gel.

**Table 1:** Materials of the study. CPC: cetylpyridinium chloride

Material	Composition	Manufacturer
*Nigella sativa* oil	Pure black seed oil 100%, pressed on cold	Imtenan Co., Cairo, Egypt
Wheat germ oil	Pure oil 100%, pressed on cold	Imtenan Co., Cairo, Egypt
CPC gel	CPC and lidocaine	Amoun Pharma Co., Alexandria, Egypt

Methods

Sample Size Calculation

Sample size calculation was performed using G*Power, Version 3.1.9.2 (Released 28 March 2014; Heinrich-Heine-Universität Düsseldorf, Düsseldorf, Germany). The effect size was 0.25 using an alpha (α) level of 0.05 and a beta (β) level of 0.05, i.e., power=95%; the estimated minimum sample size (n) should be at least 60 samples for all groups.

Animal Selection Criteria

Sixty adult male albino rats with an average weight of 180-200 grams were used in this investigation. All animals were acclimated for at least seven days before experimentation. The animals were divided into four groups and kept in a well-ventilated animal house. Temperature will be adjusted at 27-30°C, 12 hours natural light and 12 hours darkness. The animals were fed with dry rat pellet and allowed drinking water ad libitum.

Ulcer Induction

**Figure 1:**
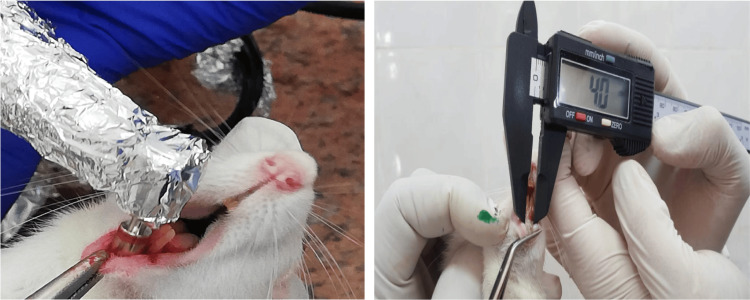
Ulcer induction on the labial mucosa corresponding to the midline between the lower two incisors of the rat, confirming the optimum width by a digital caliper.

Prior to the creation of the ulcers, all animals were anaesthetized with an intraperitoneal injection of 10% ketamine (75 mg/kg body weight) and 2% xylazine (3 mg/kg body weight). The ulcer was produced on the labial mucosa corresponding to the midline between the lower two incisors of each rat (Figure 1). For wound size standardization, punches of 4 mm in diameter, penetrating 1 ml deep in the tissue, were used [9].

Animal Grouping

After the induction of the ulcer, rats were randomly divided into four groups and were randomly allocated using computer-generated random numbers, according to the type of treatment received, as follows: Group A (control group): 15 rats remained without treatment; Group B (CPC and lidocaine oral gel group): 15 rats received a daily topical application of 1 ml of CPC oral gel painted by a brush covering the whole area of the ulcer; Group C (NS group): 15 rats received a daily topical application of 1 ml of NS oil by the same mode of application as in Group B; and Group D (WGO group): 15 rats received 1 ml of WGO, by the same mode of application as in Group B. Five animals from each group were euthanized by overdose of ether at days 3, 7, and 9.

Methods of Evaluation

Clinical Observation

The healing process of the ulcer was clinically assessed by measuring the ulcer surface area using the digital caliber at different time intervals on days 3, 7, and 9, and then tissue samples were taken for histological examination. The largest (D) and smallest (d) diameters of the induced ulcer were recorded, and the ulcer surface area was calculated using the formula A=π×D/2×d/2, where π is equal to 3.14 [10].

Histopathological Assessment

At the proposed time for each group, the labial mucosa was dissected out, fixed for 24 hours in 10% buffered formalin, and dehydrated by immersion in successive ascending concentration of ethanol. Then, they were infiltrated with molten paraffin wax (55%) to be embedded later in paraffin wax blocks. The blocks were cut with microtome to obtain sections of 4-5 μm thickness that were mounted to glass slides to be stained with hematoxylin and eosin (H&E) and Masson's trichrome stain (collagen special stain). All sections were examined under light microscope and evaluated as follows: (1) quantitatively by counting the number of inflammatory cells semi-automatically using ImageJ Fiji-64 software (National Institutes of Health, Bethesda, Maryland, United States) at magnification x400 and (2) qualitatively by observing the histological picture of tissue repair with H&E staining and collagen tissue remodeling with Masson's trichrome staining. Qualitative assessment was done according to the histological grading criteria (Table 2) [11].

**Table 2:** Histological grading criteria for healing.

Grade	Features
Grade 1 (very light healing)	Necrotic epithelium
	No collagen fibers
	Low vascularity
	No granulation tissues
	Abscess formation
Grade 2 (moderate healing)	Epithelial proliferation in the margin of ulcer
	Moderate collagen fibers
	Moderate vascularity
	Onset of granulation tissue formation
Grade 3 (advanced healing)	Epithelium continuation
	Well-organized granulation tissue
	Abundant collagen and capillaries content
Grade 4 (well-organized healing)	Complete epithelialization
	Fibrous connective tissue
	Normal amount of capillaries
	Absence of granulation tissue

Statistical Analysis

The clinical and histological results were statistically analyzed as follows: (a) First, descriptive statistics were calculated in the form of mean±standard deviation (SD), range (max-min), and coefficient of variance (CV %). b) Next, two-way analysis of variance (ANOVA) was used to compare between groups the times and their interaction for each variable under study. To further compare between subgroups, Tukey's honestly significant difference (HSD) post-hoc test was performed at a significance level of p≤0.05 or highly sign p≤0.001. c) Lastly, paired t-test was used for the intergroup comparison of the four groups.

## Results

Clinical observations

Table 3 presents a comparison between the studied groups regarding surface area over time. The studied groups include the control, NS oil, CPC gel, and WGO groups. The difference between the different groups was performed using one-way ANOVA, as presented on the table below.

**Table 3:** Comparison between the studied groups regarding surface area over time. CPC: cetylpyridinium chloride; SD: standard deviation; HSD: Tukey's honestly significant difference; P1: difference between the control and *Nigella sativa* groups; P2: difference between the *Nigella sativa* and CPC oral gel groups; P3: difference between the CPC oral gel and wheat germ oil groups; P4: difference between the control and CPC* *oral gel groups; P5: difference between the control and wheat germ oil groups; P6: difference between the *Nigella sativa* and wheat germ oil groups; F: one-way ANOVA test; ANOVA: analysis of variance; *p<0.05: statistically significant; **p≤0.001: statistically highly significant

Area	Groups				Test	
	Control	*Nigella sativa* oil	CPC oral gel	Wheat germ oil	F	P
	Mean±SD	Mean±SD	Mean±SD	Mean±SD		
Day 3	12.199±0.448	10.656±1.023	11.557±0.261	11.195±1.07	5.845	P=0.002*
HSD	P_1_=0.002*	P_2_=0.024*	P_3_=0.636	P_4_=0.39	P_5_=0.073	P_6_=0.299
Day 7	11.095±0.183	4.748±1.908	7.172±1.824	2.584±0.452	37.692	<0.001**
HSD	P_1_<0.001**	P_2_=0.005*	P_3_<0.001**	P_4_<0.001**	P_5_=0.011*	P_6_=0.013*
Day 9	9.909±0.414	1.131	2.836		187.58	0.052

On day 3, there is a statistically significant difference between the studied groups regarding the mean of ulcer surface area. When post-hoc analysis was used, the results showed that there is a significant difference between the NSgroup and each of the control (p1=0.002) and CPC oral gel (p2=0.024) groups (lowest mean of ulcer surface area in the NS group). On the other hand, there is a non-significant difference between the CPC oral gel and the WGO nor the control groups. Also, there is a non-significant difference between the WGO and any other group.

On day 7, there is a statistically significant difference between the studied groups regarding the mean of ulcer surface area. When post-hoc analysis was done, the difference is significant between each two individual groups where the least mean of ulcer surface area was present in the WGO followed by NS and then CPC oral gel and lastly control group.

On day 9, there is a statistically non-significant difference between the studied groups regarding the mean of ulcer surface area. One rat within the NS group still had unhealed ulcer and one within the CPC oral gel group versus five rats in the control group, while the mean of ulcer surface area was least in the rat treated with NS followed by that of CPC oral gel and then those within the control group. All rats of the WGO group had healed ulcers on the ninth day.

There is a statistically non-significant difference between the studied groups regarding the complete clinical healing of ulcer in rats that survived till the ninth day. All survivors within the WGO group and 80% of the survivors within both the NS and CPC oral gel groups had complete healed ulcer, while all rats of the control group still had unhealed ulcer yet without a statistically non-significant difference.

Table 4 presents a comparison between the studied groups regarding the complete clinical healing of ulcer on the ninth day with difference between groups using the chi-squares test and Monte Carlo test.

**Table 4:** Comparison between the studied groups regarding surface area over time. CPC: cetylpyridinium chloride; MC: Monte Carlo test; P: p-value

Ulcer at the ninth day	Groups				Test	
	Control	CPC oral gel	*Nigella sativa* oil	Wheat germ oil	F	P
	N=5 (%)	N=5 (%)	N=5 (%)	N=5 (%)		
Healed	0 (0%)	4 (80%)	4 (80%)	5 (100%)	MC	0.118
Traumatized	5 (100%)	1 (20%)	1 (20%)	0 (0%)		

Histopathological findings

Quantitative Analysis (Inflammatory Cell Count)

The histopathological findings including inflammatory cell count were presented in Table 5.

**Table 5:** Comparison between the studied groups regarding inflammatory cells over time. CPC: cetylpyridinium chloride; SD: standard deviation; P1: difference between the control and *Nigella sativa* groups; P2: difference between the *Nigella sativa* and CPC oral gel groups; P3: difference between the CPC oral gel and wheat germ oil groups; P4: difference between the control and CPC oral gel groups; P5: difference between the control and wheat germ oil groups; P6: difference between the *Nigella sativa* and wheat germ oil groups; F: F-ratio, i.e., one-way ANOVA test; ANOVA: analysis of variance; HSD: Tukey's honestly significant difference; *p<0.05: statistically significant; **p≤0.001: statistically highly significant

Inflammatory cells	Groups				Sign. test	
	Control group	*Nigella sativa* oil	CPC oral gel	Wheat germ oil group		
	Mean±SD	Mean±SD	Mean±SD	Mean±SD	F	p
Day 3	285.5±20.65	141.67±8.37	223.33±22.78	120.07±6.54	242.8	<0.001**
HSD	P_1_<0.001**	P_2_<0.001**	P_3_<0.001**	P_4_<0.001**	P_5_=0.001**	P_6_=0.002*
Day 7	200.75±29.17	76.1±18.03	145.2±4.47	53±12.52	124.8	<0.001**
HSD	P_1_<0.001**	P_2_<0.001**	P_3_=0.001**	P_4_<0.001**	P_5_=0.011*	P_6_=0.011*
Day 9	134.5±10.61	22.8±3.56	64±0.71	34.2±2.63	432.6	<0.001**
HSD	P_1_<0.001**	P_2_<0.001**	P_3_<0.001**	P_4_<0.001**	P_5_=0.001**	P_6_=0.011*

On day 3, there is a statistically significant difference between the studied groups regarding inflammatory cell count. On doing post-hoc analysis, the difference is significant between each two individual groups where the least count was present in the WGO followed by NS and then CPC oral gel and lastly control group.

On day 7, there is a statistically significant difference between the studied groups regarding inflammatory cell count. On doing post-hoc analysis, the difference is significant between each two individual groups where the least count was present in the WGO followed by NS and then CPC oral gel and lastly control group.

On day 9, there is a statistically significant difference between the studied groups regarding inflammatory cell count. On doing post-hoc analysis, the difference is significant between each two individual groups where the least count was present in the WGO followed by NS and then CPC oral gel and lastly control group.

Qualitative Analysis

Histological analysis of the labial mucosa at the site of ulceration revealed different histopathological pictures with various healing grading scores along different time intervals of the experiment.

H&E Staining Results

**Figure 2:**
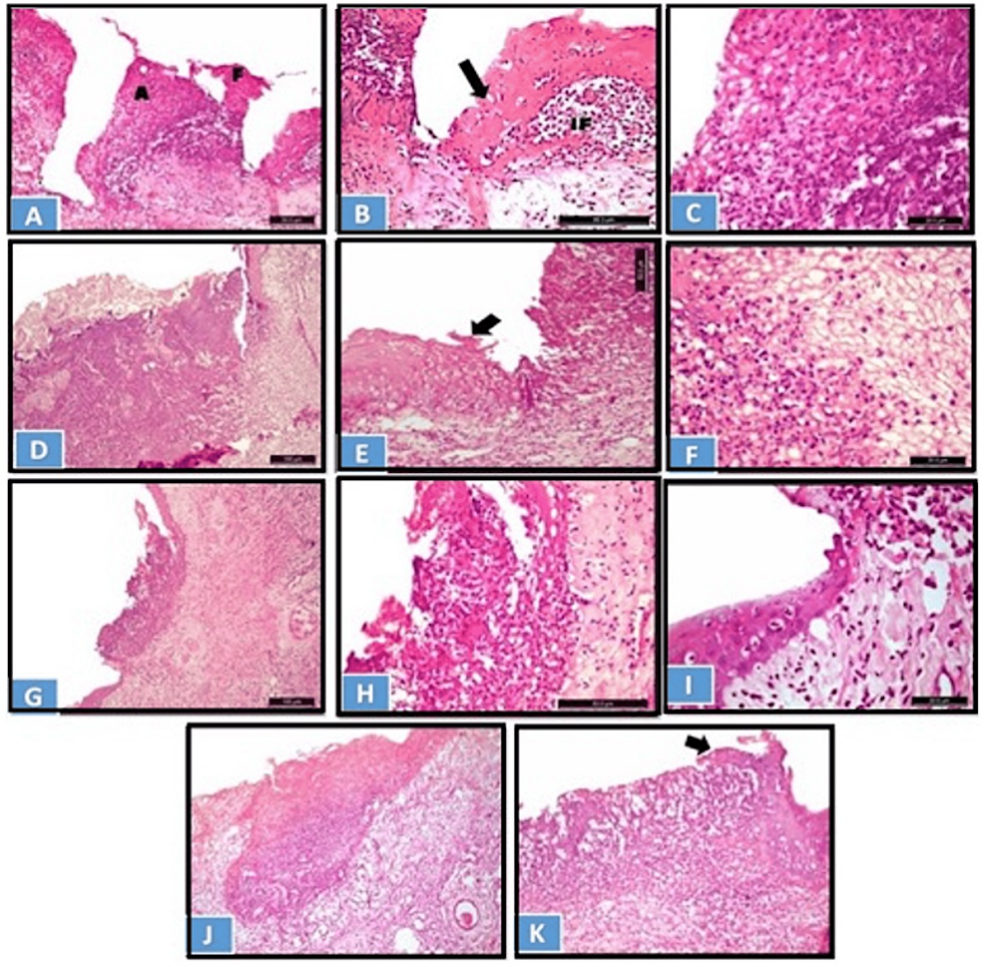
Photomicrograph of the ulcer site on day 3: (A-C) control group, (D-F) CPC-treated group, (G-I) NS-treated group, and WGO-treated group (J and K). A) Epithelial loss, abscess formation (A), and protruded necrotic tissue admixed with massive inflammatory cells, in addition to necrotic epithelial fragmentation (H&E x200). B) Necrotic epithelium at the wound margin with inflammatory cell invasion and subepithelial heavy IF (H&E x400). The black arrow shows the necrotic epithelium at the would margin with inflammatory cell invasion. C) Hyperemic zone composed of necrotic coagulative tissue mixed with PMNL infiltrate (H&E x630). D) Wide zone of coagulative tissues and necrotic epithelium at the border (H&E x100). E) Necrotic lateral epithelium (arrow) and massive inflammation in the subepithelial lamina propria (H&E x200). The black arrow shows the necrotic lateral epithelium and massive inflammation in the subepithelial lamina propria. F) Coagulative hyperemic exudate mixed with heavy PMNL inflammatory cell infiltrate (H&E x630). G) Localization of the hyperemic zone and the inflammatory infiltration to the ulcer site (x100). H) Higher magnification to the previous figure (x400). I) Lateral epithelial migration at the subepithelial lamina propria with moderate IF (H&E x630). J) Localized coagulative tissue formation, with an increase in the vascularity of the underlying lamina propria (H&E x100). K) The black arrows shows the epithelial migration at the lateral border of the ulcer site (arrow) (H&E x200). CPC: cetylpyridinium chloride; NS: *Nigella sativa*; WGO: wheat germ oil; IF: inflammatory infiltrate; PMNL: polymorphonuclear leukocyte; H&E: hematoxylin and eosin

Figure 2 shows a photomicrograph of the ulcer site on day 3 with various healing grading scores.

On day 3, the control and CPC oral gel-treated groups showed criteria of grade 1 healing, while the WGO and NS groups showed criteria of grade 2 healing according to the histological grading criteria [11]. In the control group, the wound area showed complete loss of surface epithelium, and the epithelial lining at the wound border appeared edematous and invaded by inflammatory cells. The area of ulceration showed clotted blood and protruded necrotic coagulated mass admixed with heavy inflammatory cell infiltrate mainly composed of neutrophils, macrophages, and lymphocytes (Figure 2 A-C). In the CPC oral gel-treated group, the wound area showed complete loss of surface epithelium and necrotic epithelial lining at the border. A wide area of hyperemic zone composed of necrotic coagulation tissue and clotted blood filled the wound area, mixed with heavy inflammatory cell infiltrate (Figure 2 D-F). In the NS-treated group, the ulcerated area showed loss of epithelial lining; however, migration of epithelial lining started from the lateral sides. Necrotic mass of coagulation mixed with inflammatory cell infiltrate was localized to the ulceration area. The underlying lamina propria showed moderate amount of collagen fiber formation (Figure 2 G-I). In the WGO-treated group, the ulcerated area showed destruction of epithelium lining; however, the lateral sides showed epithelial migration. Endophytic necrotic mass of coagulation mixed with inflammatory cell infiltrate were limited to the ulceration site (Figure 2 J and K).

**Figure 3:**
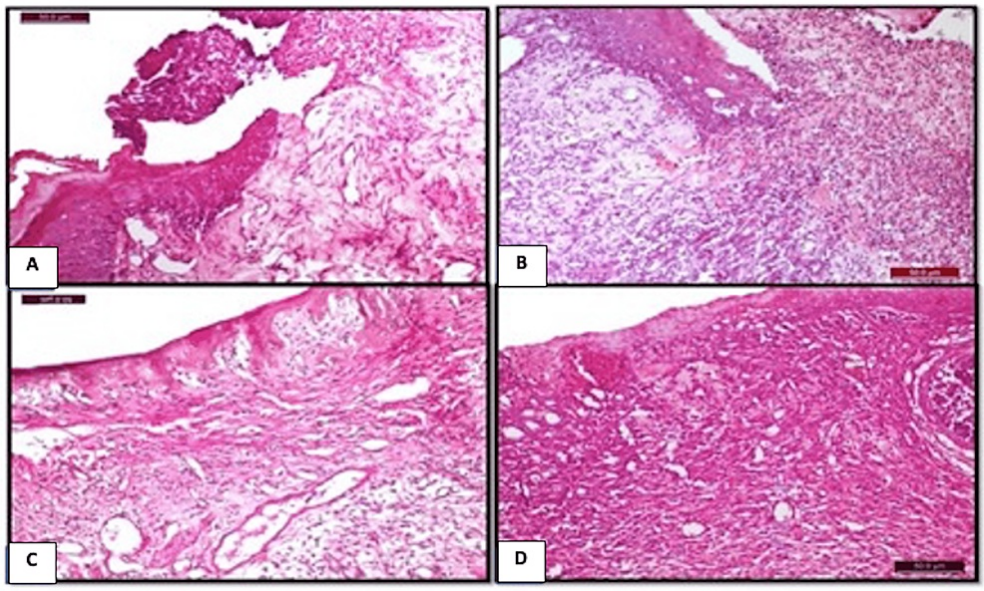
Photomicrograph of the different groups at day 7 post-ulceration induction. A) Control group: showing epithelial proliferation at the lateral side of the ulcer site (H&E x400). B) CPC-treated group: showing coagulative mass mixed with PMNLs protruded from the wound site and epithelium proliferation at the lateral border (H&E x100). C) NS-treated group: showing the formation of thin epithelial tissue covering the ulcerative site and the subepithelial connective tissues composed of well-organized granulation tissues with abundant vascularity (H&E x200). E) WGO-treated group: showing proliferated epithelial tissue covering the ulcerative site, with the formation of heavily vascularized granulation tissues in the subepithelial connective tissues (H&E x100). CPC: cetylpyridinium chloride; PMNLs: polymorphonuclear leukocytes; NS: *Nigella sativa*; WGO: wheat germ oil; H&E: hematoxylin and eosin

Figure 3 shows a photomicrograph of the different groups at day 7 post-ulceration induction.

On day 7, the control and CPC oral gel-treated groups showed criteria of grade 2 healing, while groups treated with WGO and NS showed criteria of grade 3 healing. In the control and CPC-treated groups, the wounds of the control group displayed epithelial migration, and a thin epithelial rim begins to extend at the wound periphery (Figure 3 A and B). In the WGO-treated group, epithelialization proliferation is recognized. In the NS-treated group, partial epithelial coverage was formed at the wound area (Figure 3 C and D).

**Figure 4:**
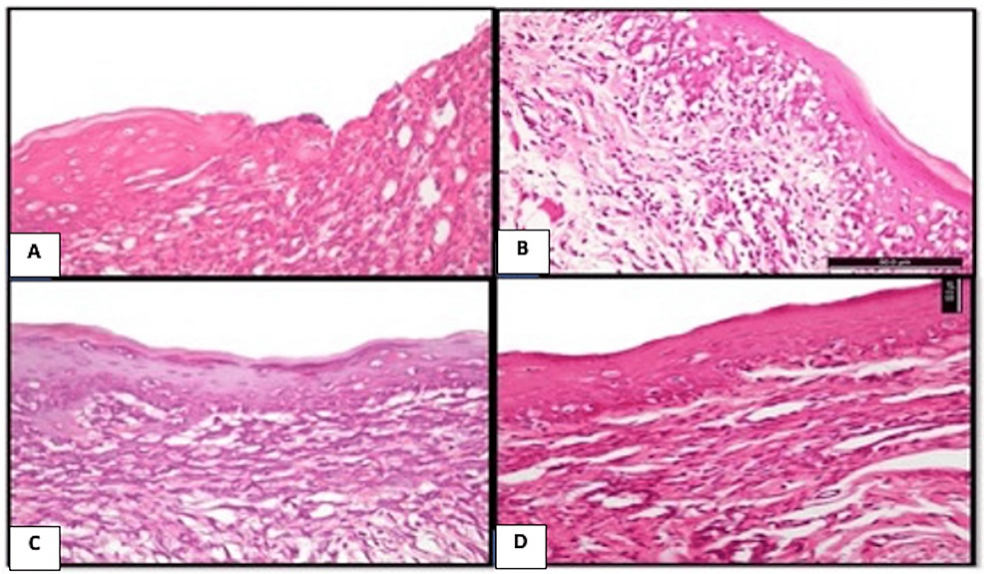
Photomicrograph at day 9 post-ulcer induction. A) Control group: showing incomplete closure of the wound site and increased mitotic activity in the epithelium in addition to inflamed subepithelial connective tissues. B) CPC oral gel-treated group: showing closure of the ulcer site with atrophied vacuolated epithelium. C) NS-treated group: showing complete closure of the ulcerated site with normal epithelial lining. D): WGO-treated group: showing complete closure of the ulcerated site with normal epithelial lining (H&E x200). CPC: cetylpyridinium chloride; NS: *Nigella sativa*; WGO: wheat germ oil; H&E: hematoxylin and eosin

Figure 4 shows a photomicrograph at day 9 post-ulcer induction.

On day 9, the control group showed criteria of grade 2 healing (moderate healing). In the control group, the ulcerated area was incompletely covered by epithelial tissues (Figure 4 A). The CPC oral gel-treated group showed criteria of grade 3 healing (advanced healing) according to the histological grading criteria. The ulcerated area was completely covered but with atrophic epithelial lining (Figure 4 B). Groups treated with WGO and NS showed well-organized healing in the form of complete epithelialization (Figure 4 C and D).

Masson's Trichrome Staining Results

**Figure 5:**
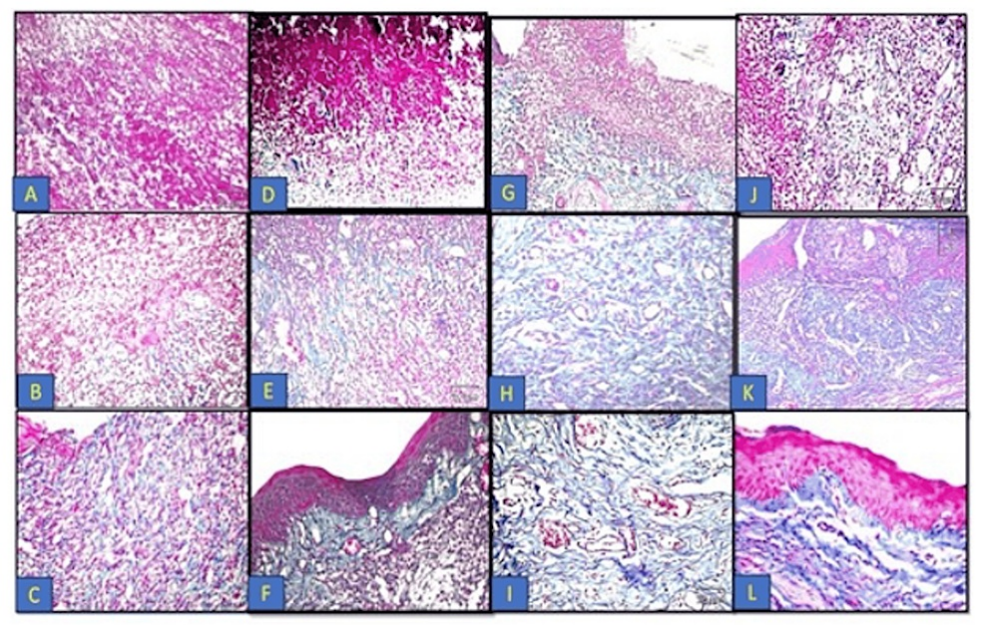
Masson's trichrome results of the different groups at different time intervals: control group (A-C); CPC-treated group (D-F); NS-treated group (G-I), and WGO-treated group (J-L). A) Control group at day 3 showing thrombic coagulative exudate admixed with PMNL infiltrate with a very thin collagen fiber formation (x200). B) Day 7 post-ulceration induction displayed heavily inflamed granulation tissue (x200). C) Day 9 post-ulcer induction showing inflamed subepithelial connective tissues (x100). D) CPC-treated group at day 3 showing very thin collagen fibers mixed with PMNL infiltrate (x100). E) Day 7 post-ulceration induction displayed collagen fiber formation in addition to inflamed granulation tissues (x200). F) Day 9 post-ulceration induction displayed the formation of collagen fibers in the underlying lamina propria in addition to the presence of granulation tissues (x200). G). NS-treated group at day 3 showing new collagen fibers beneath the ulcerated wound area (x100). H) At day 7 showing the formation of a well-organized granulation tissue, collagen fibers, and new blood capillaries (x400). I) At day 9 showing well-organized fibrous tissues composed of collagen bundles which appeared in blue color, associated with fibroblasts in addition to blood capillaries (x400). J) WGO-treated group at day 3 showing new collagen fiber and new blood capillary formation beneath the ulcerated wound area (x200). H) At day 7 showing the formation of well-organized granulation tissues and mature collagen fibers (x100). I) At day 9 showing dense fibrous tissues composed of collagen bundles which appeared in blue color, associated with fibroblasts in the lamina propria (Masson's trichrome x400). CPC: cetylpyridinium chloride; NS: *Nigella sativa*; WGO: wheat germ oil; PMNL: polymorphonuclear leukocyte

Figure 5 shows Masson's trichrome results of the different groups at different time intervals.

On day 3, the control group showed few collagen fiber formation admixed with the polymorphonuclear leukocyte (PMNL) infiltrate found in the underlying lamina propria. The CPC oral gel-treated group showed very thin collagen fibers were formed, distributed haphazardly admixed with the inflammatory cells. In the NS group, the subepithelial region showed the formation of new thin collagen fibers, accompanied with new blood capillaries. The WGO group showed the formation of new collagen fibers in the lamina propria underlying the ulcerated site.

On day 7, in the control and CPC-treated group, the underlying lamina propria beneath the ulcerated area appeared as a heavily inflamed granulation tissue. In the NS group, the subepithelial lamina propria showed a well-organized granulation tissue with abundant mature collagen fibers. In the WGO group, the underlying lamina propria showed fibroplasia with active fibroblast and new blood vessel formation, and moderate inflammatory infiltration appeared.

On day 9, in the control group, underlying connective tissues showed granulation tissues admixed with inflammatory cell infiltrate. The CPC-treated group revealed the collagen fiber formation in the underlying lamina propria in addition to the presence of granulation tissues. In the WGO and NS groups, underlying connective tissues showed dense fibrous tissue composed of collagen bundles and fibroblast cells deposited horizontally below the epithelium surface, in addition to the absence of granulation tissue.

## Discussion

Oral ulceration is a commonly presenting sign of a wide spectrum of diseases of the oral cavity in children involving many etiologic factors. These lesions may pose a unique diagnostic challenge for clinicians due to the overlap of clinical and histological features between different types of ulcerated lesions [12].

Wound healing is a natural physiological reaction to tissue injury. However, wound healing is not a simple phenomenon but involves a complex interplay between numerous cell types, cytokines, mediators, and the vascular system. The cascade of initial vasoconstriction of blood vessels and platelet aggregation is designed to stop bleeding followed by an influx of a variety of inflammatory cells, starting with the neutrophil. Inflammatory cells, in turn, release a variety of mediators and cytokines to promote angiogenesis, thrombosis, and re-epithelialization. The fibroblasts, in turn, lay down extracellular components which will serve as scaffolding [13].

This discussion focused on several modes of action by which different materials promote wound healing qualities and parameters. It is worth noting that topical therapy is often used as a therapeutic approach in wound management. In ulcer treatment, topical application is often used as a therapeutic method. The use of a natural product in the management of ulcer healing has been proposed [5,14]. Therefore, this study evaluated and compared, clinically and histologically, the ability of NS and WGO versus conventional treatment (CPC oral gel) in promoting the healing of induced traumatic ulcer in albino rats.

Numerous researchers have conducted extensive studies on NS, exploring a broad range of its pharmacological activities, including its analgesic, antibacterial, anti-inflammatory, and antioxidant characteristics. NS is one of the best-ranked evidence-based herbal remedies because of its therapeutic miracles. It also reveals that thymoquinone, a significant bioactive component of the essential oil, is responsible for most of the plant's medicinal qualities [4,5,15]. Applying thymoquinone topically to wounds is an effective approach because it allows for direct penetration to the affected areas [16].

In recent research conducted by Hijazy et al. [17], the effects of oral administration on the development of edema, oxidative stress, and inflammation in mice that had paw edema were investigated. Thymoquinone has been shown to reduce pro-inflammatory mediators in inflamed paw tissue, including IL-1, TNF-α, IL-6, MCP-1, C-reactive protein, myeloperoxidase, and NF-κB. In addition, thymoquinone treatment in mice resulted in significant decreases in cyclooxygenase-2 and its product prostaglandin E2 as well as the immune reaction of TNF-α. Histopathological analysis further confirmed the antiedematous and anti-inflammatory effects of thymoquinone in inflamed tissues. The authors concluded that the results support the potential use of thymoquinone to alleviate acute inflammation due to its strong antioxidant and anti-inflammatory properties in inflamed paw tissue.

The vitamin E, unsaturated fatty acid, and other antioxidant constituents of WGO make it the best option for restoring the collagen of the skin connective tissues and help in the treatment of skin lesions and aging [18]. WGO is an ideal source of tocopherols, tocotrienols, and phenol compounds with strong anti-inflammatory and antioxidant effects as evidenced by the low O2- production and the activity of NADPH oxidase [19].

Considering the fact that immune cells are involved in all the phases of the wound healing process, linoleic acid can affect immune cell functions and play an important role in wound healing cascade [20]. Also, CPC, which is the main ingredient in CPC oral gel, has a strong antimicrobial activity. However, it has been shown to have a cytotoxic effect against human gingival fibroblasts and endothelial cells besides its antimicrobial activity against fungi and a wide range of gram-negative and gram-positive bacteria [21].

Albino rats were chosen as experimental models in this research because they certainly represent an advantage in terms of cost and ease of performance (housed, bred, and handled). Also, they have a long life span, and remarkable changes occur when no other diseases are apparent. Moreover, rats share many pathologic characteristics with humans. Male rats were chosen in this study to prevent hormonal influence on tissue metabolism and regeneration [22].

The most common small animal model utilized in trauma research is the albino rat, with the goal of advancing and developing a fundamental comprehension of the intricate posttraumatic inflammatory and regeneration pathways. Moreover, rats have thinner dermis and epidermis than humans, which might make suturing them difficult [23].

Interestingly, rats are frequently used as a special model for a part of the complex system of wound healing in humans. Wound contraction is considered to be the primary healing method of rats as opposed to re-epithelialization seen in humans [22]. Therefore, in the present study, the ulcer was induced on the labial mucosa between the lower two incisors of each rat, and for wound size standardization, punches of 4 mm in diameter, penetrating 1 ml deep in the tissue, were used [9]. To assess the healing process of the ulcer, it was clinically assessed by measuring the ulcer area using the digital caliber at different time intervals [24].

The proliferative phase occurs concurrently with the inflammatory phase and is characterized by extracellular matrix formation, angiogenesis, collagen synthesis, and epithelial proliferation, which are followed by tissue remodeling and scarring formation. The inflammatory phase is marked by the infiltration of neutrophils, macrophages, and lymphocytes. The development of granulation tissue inside the wound characterizes this phase of proliferation, which lasts from day 3 to day 14. The granulation tissue is a mixture of newly formed capillaries from the collagen, fibronectin, and hyaluronic acid matrix, as well as fibroblasts and inflammatory cells. Periodically, the number of fibroblasts declines during the maturation phase, which is followed by the regrowth of new collagen fibers and the development of the vascular system [25,26]. Fibroblasts move toward the wounded location during wound healing, producing collagen to enhance tissue permeability. This is why collagen production is crucial to the healing process [27,28].

In this study, a histological evolution was made quantitatively by counting the number of inflammatory cells semi-automatically using ImageJ Fiji-64 software at magnification x400. Histological evaluation was done in the present study by observing the histological picture of tissue repair with H&E staining and collagen tissue remodeling with Masson's trichrome staining.

Hence, at day 3, the control group and CPC oral gel group showed criteria of grade 1 healing, the wound area showed complete loss of surface epithelium, epithelial lining at the border appeared edematous and invaded by inflammatory cells, and clotted blood and protruded necrotic coagulation admixed with heavy inflammatory cell infiltrate mainly composed of neutrophils, macrophages, and lymphocytes filled the area of ulceration. Tissue sections stained with Masson's trichrome showed few collagen fibers were found.

Our findings agree with Al-Zamily and Al-Temimi's [29] study who found that the control group three days postoperatively showed a high amount of inflammatory cell infiltration with mild fibroblasts and granulation tissue. All these findings explain the introgression of ulcer healing in the control group and CPC oral gel group clinically in the present study.

Histological analysis of the labial mucosa at the site of ulceration in the NS- and WGO-treated groups on day 3 showed epithelial lining destruction; however, migration of epithelial lining started from the lateral sides. Necrotic mass of coagulation mixed with inflammatory cell infiltrate are localized to the ulceration area. The underlying lamina propria showed moderate amount of collagen fiber formation.

These findings agreed with Al-Zamily and Al-Temimi [29] who reported that three days after ulcer induction and treatment with NS oil, a moderate inflammatory cell and a mild fibroblast plug initiate the re-epithelization. Thymoquinone, the primary component of the essential oil, is primarily responsible for the biological ability of NS seeds. The seeds' oil possesses antibacterial, analgesic, antipyretic, anti-inflammatory, and antitubercular properties [15].

Also, Marchwińska and Michocka [30] demonstrated that WGO had an antimicrobial activity against *Streptococcus epidermidis* and *Pseudomonas aeruginosa*. These organisms were reported as a regular cause of postoperative infection, and this action could accelerate the ulcer and wound healing.

At day 7 follow-up, the wounds of the control group displayed epithelial migration, a thin epithelial rim begins to extend at the wound periphery, and the underlying lamina propria beneath the ulcerated area has a heavy inflamed granulation tissue. These findings were in agreement with Ali [31] who observed, seven days after ulcer formation in the rat labial mucosa, a considerable reduction in inflammatory cell infiltration with intermittent chronic inflammatory cells, an incompletely epithelized wound surface, and a modest rise in the number of blood vessels. Al-Zamily and Al-Temimi [29] found that the line of incision seven days postoperatively of the control group showed hyperplasia of the epithelial cells with round and thick rete ridge.

The CPC oral gel-treated group at day 7 showed epithelial proliferation at the lateral border with marked decrease in surface ulcer area with progress in healing. These results were in agreement with Rasic et al. [32] who demonstrated a clear benefit of CPC and lidocaine in the local treatment of induced oral mucositis and its superiority over compounded medication for local use for the same purpose. Response was marked as early as on the seventh day and then became more pronounced.

Regarding the histological results of groups treated with NS and WGO, they showed criteria of grade 3 healing according to the Shafer criteria at seven-day interval. On day 7 evaluation, the NS-treated group showed advanced healing, and partial epithelial coverage was formed at the wound area. The underlying lamina propria showed fibroplasia with active fibroblasts, new blood vessel formation, and moderate inflammatory infiltration.

In agreement with the present results study, Al-Zamily and Al-Temimi [29] found that the duration of the inflammatory phase decreased in the NS oil group compared with the control group. The collagen density and the arrangement were significantly better in the NS oil group than in the control group, and these explain the clinical reduction in the surface area of induced traumatic ulcer at this time interval in our study.

In the WGO-treated group, epithelialization proliferation was recognized with the subepithelial lamina propria showing a well-organized granulation tissue with abundant mature collagen fibers. Also, that was confirmed with the clinical observations in the present study, it showed a significant reduction in the wound area, and this was in accordance with Zakaria et al.'s [8] results, who monitored the progress in the healing of the animal's wounds treated with WGO through a photographic representation of the wound skin. For successive wound healing, photographs were taken once per week to confirm the reduction in wound diameter and to demonstrate the potential application of the WGO formulation in the treatment of animal wounds.

Clinical observation of the control group shows incomplete ulcer healing at day 9 follow-up, as the wound area was incompletely covered by epithelial tissues. Underlying connective tissues showed granulation tissues admixed with inflammatory cell infiltrate. These findings were in accordance with Russell [33] who reported a decrease in size of the wounds in rats of the control group, a little number of macrophages and neutrophils in the connective tissue, and an increase in the wound contraction and epithelization at 10-14 days after wounding.

On day 9, the CPC oral gel group showed criteria of grade 3 healing (advanced healing) according to the Shafer criteria, and clinically, it showed complete ulcer healing. In line with our study, Mehrabani et al. and Paley [34,35] proved that CPC shows a broad-spectrum antimicrobial activity and might also represent a promising antibacterial agent for the treatment of chronic wounds. This also goes with fact that the antimicrobial effect of CPC results from its ability to disrupt bacterial metabolism, inhibit cell growth, and induce cell death after the initial penetration of cell membranes and CPC is a safe antimicrobial agent for preventing biofilm formation.

Also, on day 9, the NS-treated group showed healing (well-organized healing) and also complete clinical-induced ulcer healing which agrees with Aljabre et al. [14] who had revealed that the oil obtained from NS can heal skin wounds in a rat model and the topical appliance of oil equipped from its seeds can accelerate wound healing and also the fact that the increase of blood flow to the area of ulcers and the decrease of inflammatory cells are in proportion to the reduction of the infection on the ulcer and promote the healing of ulcers [36].

At day 9 follow-up, the WGO group showed a well-organized healing in the form of complete epithelialization with the formation of collagen bundles and fibroblast cells, in addition to the absence of granulation tissue and clinically complete ulcer healing, and this goes with the fact that the vitamin E, unsaturated fatty acid, and other antioxidant constituents of WGO make it the best option for restoring the collagen of the skin connective tissues and help in the treatment of skin lesions and aging [37].

Conferring to Cavalcante et al. [10], the cicatrization process, similar to what happens in any inflammatory process, consists of steps with a sequence of alterations that can be observed throughout the period of tissue repair. The role of growth factors in the migration, proliferation, and differentiation of epithelial cells, the formation of connective tissue, and the growth of new vessel is of great importance. Thus, the proposed model is similar to human traumatic ulcers regarding both the pathological characteristics and the cicatrization mechanism. These ulcerations represent inflammatory lesions that are extremely symptomatic that also impair feeding and the patient's quality of life.

A major advantage of using animal models of wound healing is the ability to harvest tissue for histological observation. Briefly, a portion of various sizes depending on the wound area created initially is excised from the euthanized animal model and fixed prior to serial tissue processing, followed by a paraffin blocking process [38].

The current study showed that NS, WGO, and CPC oral gel treatment have beneficial effects in the inflammatory, proliferation, and maturation phases of wound healing, compared with their control groups. The maturation or remodeling phase is the last and longest phase of ulcer wound healing. The most important development is the remodeling and maturation of collagen during this phase.

Compared with their control groups, the proliferation phase was positively affected in the treatment groups by the increase in the number of fibroblasts as well as the stimulation of the collagen synthesis and composition. Compared with the NS oil and CPC oral gel treatment, the WGO proved more effective in the proliferation phase with complete ulcer healing in all animals treated with WGO in the present study.

Furthermore, Ghafoor et al. [39] mentioned that the frequent use of the quaternary ammonium compound CPC could likewise result in bacterial drug resistance. Thus, use of herbal treatment modalities like NS and WGO to promote the healing of oral ulcers became the new era.

One of the limitations of the present study is that very little or no similar researches were found on our topic especially the histological part in comparing the effect of NS or WGO to CPC on induced traumatic ulcers in rats in the literature. In addition, this study didn't assess the cellular biomarkers of the healing process which is considered as another limitation of the current study.

## Conclusions

WGO was significantly more effective in the treatment of animal-induced ulcer compared to NS oil or CPC oral gel. The present study supported that the use of complementary and alternative medicines such as WGO and NS oil produced ameliorative effects with variable degree on induced traumatic ulcer in albino rats as they successfully revert the traumatized tissue into a nearly normal condition.
